# Postanesthetic Severe Oral Angioedema in Patient's Taking Angiotensin-Converting Enzyme Inhibitor

**DOI:** 10.1155/2014/693191

**Published:** 2014-11-06

**Authors:** Acílio Marques, Carla Retroz-Marques, Sara Mota, Raquel Cabral, Matos Campos

**Affiliations:** Anaesthesiology Department, Coimbra University Hospital Center, Praceta Mota Pinto, 3001-301 Coimbra, Portugal

## Abstract

Angiotensin-converting enzyme (ACE) inhibitors are the leading cause of a drug-induced angioedema. This occurrence is frequently underdiagnosed, but its relapse can be life-threatening. The authors' intention in reporting this clinical case is to sound a warning about reviewing attitudes and surveillance to try to improve patient perioperative safety.

## 1. Introduction

Angiotensin-converting enzyme (ACE) inhibitors are widely prescribed and are the leading cause of a drug-induced angioedema [1]. However, contrary to what may happen with other drugs, this adverse reaction is frequently missed because it can start years after beginning the treatment and recurs erratically but with increased morbidity severity or even mortality [2].

The management of these severe adverse reactions has been discussed but still missing consensus about perioperative surveillance guidelines in patients taking ACE inhibitors [3–6]. However, it is obviously unquestionable that the quality of perioperative care is crucial to the patient's safety, and all medical surveillance decisions must be carefully planned and implemented.

The severity of this case legitimizes it being reported to raise the awareness of health care professionals and propose preventive attitudes for discussion that could improve the perioperative safety of patients.

The patient reviewed the case report and gave permission for the authors to publish.

## 2. Case Description

We report the case of a male Caucasian, 81-year-old, weighing 90 kg, and 175 cm tall. He was hospitalized in the Burn Intensive Care Unit (BICU) with a third-degree burn of the foot and he was proposed for surgical cleaning with skin grafting. The patient was conscious and oriented but with amnesia regarding his medical history. The anesthetic risk by the American Society of Anesthesiologists classification was grade III due to hypertension, and he had NYHA class II heart failure. The usual pharmacological therapy was perindopril, furosemide, finasteride, and pantoprazole.

We performed a combined anesthesia: femoral/sciatic nerve blocks with ropivacaine associated to general anesthesia with propofol, fentanyl, and sevoflurane. For airway patency, we used a nontraumatic supraglottic device (Igel), and the patient was in spontaneous ventilation all the time. There were no abnormal or unexpected incidents during the operation. The overall perioperative period was spent in the BICU with constant medical surveillance.

**Figure 1:**
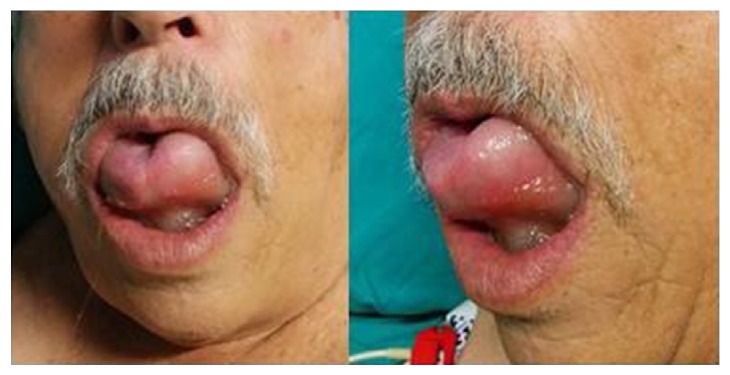
Oropharyngeal edema involving the tongue.

Five hours later, the patient developed dysphagia and mild respiratory distress. He was aware and oriented but anxious with polypnea and tachycardia. The main clinical sign was oropharyngeal edema involving the tongue (Figure 1).

The upper airways were nebulized with epinephrine. Intravenous drugs were given: 250 mg methylprednisolone and 2 mg clemastine. We opted to keep the patient under strict medical surveillance without additional specific drug therapy but keeping a possible emergency tracheostomy in mind. The daily therapy was reviewed, and the ACE inhibitor, perindopril, was suspended. Laboratory blood levels of IgE and tryptase were normal.

**Figure 2:**
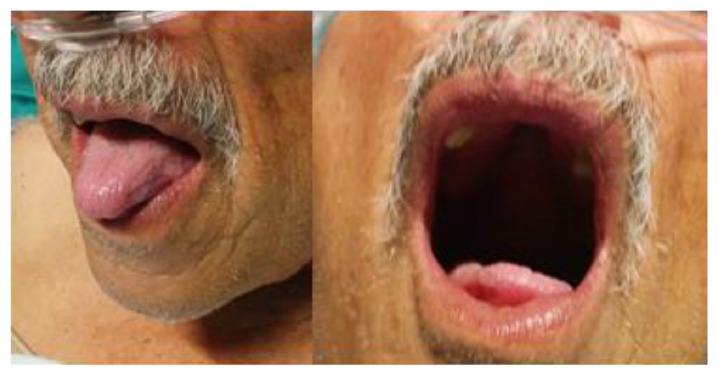
After 24 hours of ACE inhibitor suspension.

After 24 hours of ACE inhibitor suspension there was a clinical improvement (Figure 2). There were no new episodes in the six-month follow-up period.

## 3. Discussion

ACE inhibitors are the most common cause of nonhereditary angioedema (25–39%). The probability that a patient taking an ACE inhibitor will go on to develop angioedema is 0.1–0.7% [7–9]. However and unlike other cases of drug-related angioedema, this adverse reaction is frequently missed because it can start years after beginning the treatment and recurs erratically while treatment continues. Another clinical concern is that the severity of adverse reactions increases with each recurrence and can be life-threatening [10–13].

The bradykinin receptor and its active metabolites have been demonstrated experimentally as humoral mechanisms of angioedema due to increased levels of nitric oxide, prostacyclin PG12, and neuropeptide substance P and a consequent increase in vascular permeability. The inactivation of kinins is mainly caused by angiotensin-converting enzyme (ACE), but other important enzymes are aminopeptidase (APP), dipeptidyl peptidase IV (DPP-IV), and neutral endopeptidase (NEP) [3].

Patients taking other drugs that are also bradykinin-degrading enzyme inhibitors are at increased risk. Diabetic patients have new drug therapies that are DPP-IV inhibitors (sitagliptin, saxagliptin, and vildagliptin). Transplant recipients with immunosuppressant medications should receive inhibition of DPP-IV enzyme activity to improve graft survival success [3].

In addition to the amount of bradykinin, individual sensitivity is an important factor to trigger angioedema. In the presence of clinical angioedema, we should exclude hereditary autosomal dominant disease typified by a deficiency or dysfunction of the C1-esterase inhibitor [14].

Perioperative patients taking ACE inhibitors have mainly been studied in relation to anesthetic hemodynamic stability [15, 16]. The possibility of severe angioedema must be discussed enough for the best practices improvement [17].

In perioperative medicine, preventive attitudes begin with preoperative evaluation, anesthetic-surgery planning, and appropriate postoperative recovery care [18]. These surveillance attitudes do not necessarily mean more medical care but are essential for a better health care.

In the case reported here, the first main concern was the hypothesis of an anaphylactic reaction to surgical/anesthetic procedures and the inherent therapy. However, the painless, nonpruritic mucosal edema was restricted to the oral cavity localized, with only slight response to the antianaphylactic drugs, and the blood tests were normal. The only drug discontinued was the ACE inhibitor, while the other drugs were administered daily, and there was a late but sustained clinical improvement without further episodes in the following months. We conclude that the patient had a severe life-threatening angioedema with high probability that the etiology was directly related to the previous treatment with an ACE inhibitor [7].

There are risk factors for oral angioedema that should be evaluated in patients taking ACE inhibitors: older age, femal sex, Hispanic race, or African-American ancestry. Also, patients should be screened for positive smoking history, coexistent cardiopulmonary disease, class III or above of Anesthesiologist's American Society, previous allergic reactions, and cough or taking other drugs that are also bradykinin-degrading enzyme inhibitors [19, 20].

This patient had several risk factors for severe angioedema: the therapy with ACE inhibitor which was not stopped, older age, Hispanic race, class III of Anesthesiologist's American Society, and coexistent cardiopulmonary disease.

The patient's therapy with ACE inhibitor, perindopril, was not stopped before surgery because the patient had no previous adverse reactions, and postoperatively he will stay in Burn Intensive Care Unit under safety vigilance. The ACE inhibitors intake suspension or replacement remains a dilemma. Researchers of Toronto General Hospital reviewed data from more than 61,000 perioperative patients and concluded that therapy with ACE inhibitors does not have additional morbidity or mortality. Although this evidence is encouraging, randomized prospective confirmatory trials must confirm that conclusion [21]. The American College of Physicians issued recommendations on the perioperative management of patients stating that clinicians should consider holding or reducing the usual dose of ACE inhibitors for better perioperative patient's hemodynamic stability [22, 23].

In the absence of adverse reactions, drug discontinuations have to be carefully planned and weighted [24, 25]. If patients have been recommended to discontinue ACE inhibitor and replace it, upon advice of a physician, such exchange should take place as early as possible. However, some studies revealed that patients are still at risk of developing angioedema during several months after therapeutic stop [26]. The patients with adverse reactions to ACE inhibitors can have safer alternatives with angiotensin II receptor blockers (ARBs) or calcium channel blockers [27, 28]. Although, there are reports that 10% of patients with previous ACE inhibitors angioedema also develop this adverse reaction after changing the medication to ARBs [29, 30].

The anesthetic airway manipulations are an additional risk factor for oropharyngeal edema. Although trauma may trigger angioedema, the intensity and time to onset are not clear [31]. This patient had a nontraumatic supraglottic device (Igel); however we cannot exclude this etiology for angioedema trigger. Whenever possible, avoiding airway manipulating should be one of the angioedema preventive anesthetic attitudes for patients taking ACE inhibitor.

The postoperative vigilance is the main key for the anesthetic plan and safety of these patients. The oral edema severity of this case occurred five hours after the operation. Fortunately, the patient was under medical supervision in a specialized intensive care unit. Therefore, patients with recent ACE inhibitor intake should have postoperative surveillance suited to their conscientious autonomy and accessibility to emergency care units.

Our report raises the awareness of health care professionals for preventive attitudes. Probably in the near future the pharmacogenomics and personalized medicine applications can improve patient safety and prevent morbidity [32, 33].

## 4. Conclusions

Perioperative ACE inhibitor angioedema is a rare occurrence but can be life-threatening. Unfortunately, there is no specific test that can predict that adverse reaction. ACE inhibitor intake withdrawn is the main treatment and the only prophylactic measure to avoid the drug-induced angioedema.

Preoperative risk stratification aims to determine probabilities, optimize medical therapy, and modify risk factors. Before the surgery, patients with increased risk factors for severe cases of angioedema should withdraw ACE inhibitor as early as possible.

After the ACE inhibitors withdraw, the probability of angioedema lowers with the passing time. However the occurrence of angioedema should always be adequately surveyed.
